# Genetic Susceptibility Testing and Readiness to Control Weight: Results from a Randomized Controlled Trial

**DOI:** 10.1002/oby.20958

**Published:** 2014-12-17

**Authors:** Susanne F Meisel, Rebecca J Beeken, Cornelia H M van Jaarsveld, Jane Wardle

**Affiliations:** 1 Cancer Research UK Health Behavior Research Centre, Department of Epidemiology and Public Health, University College LondonUK; 2 Department of Primary Care and Public Health Sciences, King's College LondonLondon, UK

## Abstract

**Objective:**

To test the hypothesis that adding obesity gene feedback (*FTO*) to simple weight control advice at a life stage with raised risk of weight gain (university) increases readiness to control weight.

**Methods:**

Individually randomized controlled trial comparing the effect of: (i) simple weight control advice plus *FTO* feedback (FA) and (ii) simple weight control advice only (AO) on readiness to engage with weight control. Differences in stage of change by genotype and differential weight control behaviors were secondary outcomes.

**Results:**

Of 1,016 participants randomized, only 279 completed follow-up, yielding 90% power to detect a small effect for readiness to control weight. As predicted, FA participants were more likely to be in the contemplation stage than AO participants (*P* = 0.023). Participants receiving higher-risk genetic results were at a higher stage of change than controls (*P* = 0.003), with a trend toward a higher stage of change than those getting lower-risk results (*P* = 0.051). Lower-risk results did not decrease weight control intentions compared with controls (*P* = 0.55). There were no group differences in adherence to recommended weight control behaviors (*P* = 0.87).

**Conclusions:**

Adding *FTO* feedback to weight control advice enhanced readiness to control weight, without evidence for genetic determinism, but had no more effect on behavior than weight control advice alone.

## Introduction

“Personalizing” lifestyle interventions by including information on genetic risk has been proposed as a novel way to encourage efforts at obesity prevention 1. Receiving a genetic test result indicative of increased obesity risk is expected to result in prevention efforts by increasing risk perceptions, in line with protection motivation theories 2. However, it has also been argued that genetic information could lead to disengagement with health behavior change 3,4. Receiving a lower-risk result may result in decreased risk perceptions and thus decreased motivation to prevent obesity. Furthermore, some evidence indicates that genetic information may lead to fatalism and diminish perceived control over disease development 5,6.

Although several studies have examined the psychological impact of genetic test feedback for risk of obesity in individuals already struggling with weight control and found it effective for increasing intentions to lose weight 7–9, a recent Cochrane review failed to confirm any effect on behavior change 10. However, most studies in this review focused on gene feedback to aid smoking cessation. In addition, none has investigated the effects of genetic test feedback for intentions to prevent weight gain, although the prevention of ill health is one of the main expected benefits of returning genetic test feedback to healthy individuals 1.

One period that has been associated with an increased risk of weight gain is the transition from high school to university 11,12. Although anecdotal reports of weight gain of 15 lbs in the first year of university are prevalent (the so called “Freshman 15”), empirical evidence indicates that actual weight gain is likely closer to 5 lbs 13,14. However, because students commonly have low intentions to implement healthy behaviors 15,16, weight gained during this period may not be lost over time, leading to an increased proportion of young adults classified as overweight or obese 17.

We were therefore interested in whether genetic feedback for one gene (*FTO*) that has been consistently associated with risk of weight gain 18, would increase readiness to control weight in a population of first year students, in line with the transtheoretical model of behavior change 19.

### Study objectives

#### Primary research objective

Evidence from earlier studies on genetic test feedback indicates that genetic test feedback can increase behavior change intentions regardless of the “actual” genetic test result, presumably because of its personalized nature 20–22. The primary aim of the study was therefore to test the hypothesis that adding *FTO* genetic test feedback to simple weight control advice (Feedback and Advice, FA) would result in greater readiness to control weight in the intermediate-term (1 month later) compared with weight control advice alone (Advice only, AO).

#### Secondary research objectives

All secondary objectives were exploratory because the trial was not powered to detect significant differences. There were three secondary objectives.

(i) To examine differences in the effect of *FTO* feedback on readiness to control weight in normal-weight vs. overweight/obese individuals. On the basis of previous studies we hypothesized that *FTO* feedback would have greater impact in those already overweight or obese 7–9.
(ii) To examine differences in readiness to control weight in those receiving higher-risk genetic results, lower-risk results, and no feedback. On the basis of previous research using hypothetical scenarios 21, we hypothesized that a higher-risk result (AA/AT) would result in greater readiness to control weight than lower-risk (TT) results, or no genetic feedback (controls). We also explored whether lower-risk *FTO* feedback reduced readiness to control weight compared with not receiving any feedback, to address the concern about “complacency” raised in the literature 3.
(iii) To explore whether genetic test feedback increased adherence to weight control advice. We hypothesized that FA participants would be more likely to adhere to the “tips” included in the weight control advice than AO participants.

## Methods

### Study design

**Figure 1:**
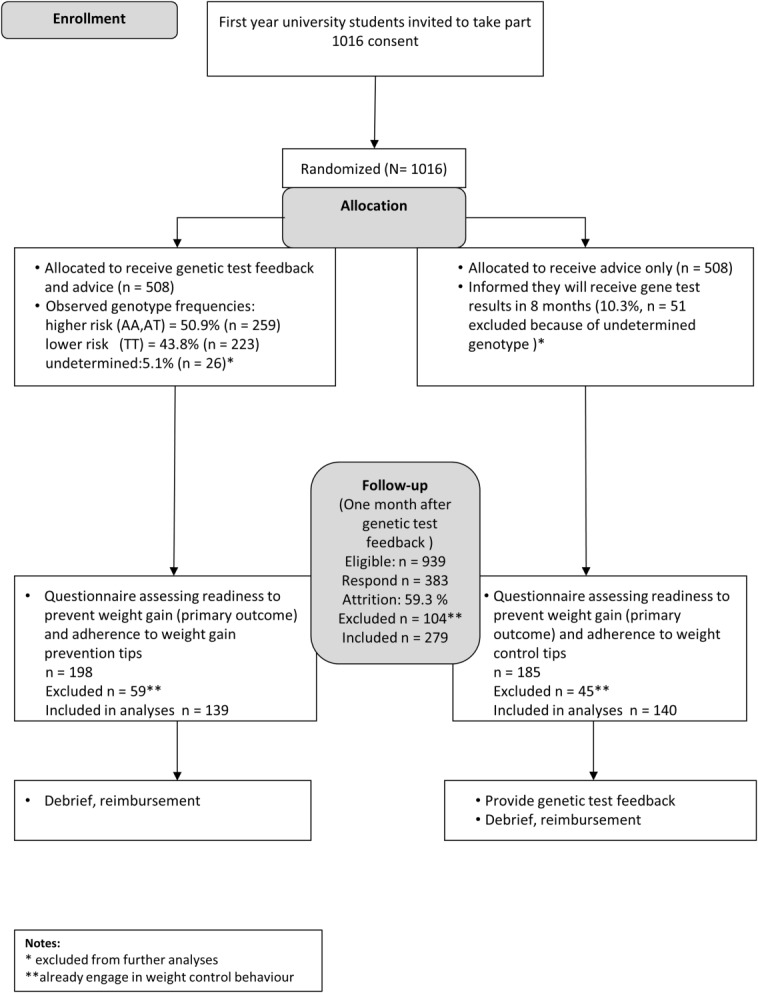
Flowchart study procedures.

The design was a single-center, open, two-arm, parallel group, individually randomized (1:1 ratio) controlled trial comparing the effect of weight control advice plus genetic test feedback (FA) with advice only (AO), on readiness to control weight (Figure 1). Ethical approval was granted by the University College London Research Ethics Committee for non-NHS research in September 2010 (Application no: 2471/003).

### Participants

A volunteer sample of 1,016 students took part in genetic testing at baseline. All students aged 18-30 years based at a large London university were invited to participate.

### Study setting

University College London (UCL) enrolls over 14,000 new undergraduates each year (mean age at enrolment: 22.8 years, 49% male, 40% international students; http://www.ucl.ac.uk/srs/statistics). Participants were recruited using email advertisements in the first 2 weeks of the first term (late September) of three consecutive academic years (2010-2013). The study ran over the course of the academic year, with follow-up one month after the intervention group received the genetic test result (February). Recruitment ended once the target number of participants was reached.

### Interventions

#### DNA collection and genotyping

Following informed consent, all participants were asked to give a saliva sample for DNA collection by placing some sugar onto their tongue to stimulate saliva flow and then spitting into a plastic tube to generate 1.5-2 ml of saliva. DNA was extracted and analyzed at the Institute of Metabolic Sciences, Cambridge, UK, as previously published 23.

#### Weight control leaflet

A weight control leaflet was developed based on a low-intensity, habit-based, weight-loss intervention that has shown promising results 24,25. It was divided into three short sections: The first section outlined why it is easy to gain weight at university, the second explained the contribution of genes to weight gain, and the third consisted of seven tips for weight control. Each tip had a memorable heading followed by a short explanation and included the following items [watch portion sizes, avoid second helpings, slow down eating, focus on your food (avoid mindless eating), pass up snacks between meals, avoid sweet drinks or choose a “lite” drink, integrate physical activity into every day].

### Intervention group (feedback and advice, “FA” group)

The FA group received the weight control leaflet with their *FTO* gene test result ∼four months after baseline data collection. The genetic test result was given in a letter in an email attachment, so that students could read it at a convenient time for them. The letter contained the personal result and brief information about the *FTO* gene, its mode of inheritance, and the magnitude of influence on body weight 7,26.

### Control group (advice only, “AO” group)

AO participants received the weight control advice leaflet attached to an email in identical format to, and at the same time as, the intervention group. They were informed that they would receive their *FTO* genetic test result by the end of the academic year, resembling a “wait-list control” group for the genetic test feedback condition.

### Measures

#### Demographic characteristics

Demographic information collected included age and gender.

#### Primary outcome

Readiness to control weight was assessed using a validated measure of readiness for behavior change 27 adapted for prevention of weight gain. Table1 shows the statements and corresponding stages of change. The time frame was adjusted to one rather than six months to reflect the time frame of the study.

**Table 1:** Stages of change and corresponding statements

Stage of change	Questionnaire item
**Precontemplation**	I am not trying to control my weight, and I have no intention of doing so in the next month
**Contemplation/preparation**	I am not trying to control my weight, but I am thinking of doing something in the next month
**Action**	I started to try to control my weight within the last month
**Maintenance**	I have been trying to control my weight for more than a month

#### Secondary outcome

Frequency of adherence to each tip was recorded on a five-point Likert scale, with response options of “never,” “occasionally,” “sometimes,” “most of the time,” and “always.”

### Sample size

A power calculation conducted a priori using GPower (version 3.1) showed that a total sample size of 251 would be sufficient to detect a small effect (*d* = 0.25) on motivation to control weight between “FA” and “AO” with ≥90% power at the 5% significance level.

### Randomization

Data were anonymized using serial numbers immediately after saliva collection. Participants were randomly assigned following simple randomization procedures to either FA or AO. Group allocation was stratified by data collection wave, before genetic test results became available. The randomization sequence was generated by SFM using the “randomize” function of the Statistical Package for Social Sciences (SPSS) version 20 (Chicago, IL) which randomly assigns a set number of cases (here: 100%) to a specified number of groups (here: 2), corresponding to a 1:1 allocation ratio of treatment and control group.

### Blinding

Participants were not blind to group allocation. However, they were made aware of group allocation only at the point at which they received either gene feedback with the advice leaflet or only the advice leaflet, minimizing the risk of bias. Furthermore, all participants only knew they were taking part in a study about genes and eating behavior and that they would be randomized; therefore, self-report responses to the primary outcome should not have been influenced. The data collector was unblinded, but questionnaire data (including the primary outcome) were collected online, and not in the presence of any member of the research team, to minimize the risk of inadvertent data manipulation. Finally, because the first author (SFM) acted as both data collector and data analyst, she was unblinded, but a data analysis plan had been drawn up prior to data collection, and decisions to change this were made with the rest of the research team who were blinded, so this knowledge is unlikely to have affected the final analysis.

### Statistical analyses

Analyses were planned to be per protocol (completers only) because of the anticipated large amount of missing data previously reported in student samples 11. Analyses were carried out using the Statistical Package for the Social Sciences SPSS v. 20 (Chicago, IL).

Differences between completers and non-completers on the outcome measures were assessed with chi-square tests for categorical variables and independent-samples *t* tests for continuous variables. As specified in the study protocol 28, participants who reported having been controlling their weight for more than 1 month were excluded from analyses (*n* = 104), because we were interested in the effect of *FTO* feedback in individuals who had not yet reached the maintenance stage of weight control. To assess effects of excluding these participants we conducted a sensitivity analysis with the full sample. No differences in results were observed (data not shown).

For the primary outcome, ordinal logistic regression (Polytomous Universal Model, PLUM) was used to assess the difference between “FA” and “AO” group in readiness to control weight. Results from secondary analyses were considered exploratory. All models included age, gender and weight status as covariates. Age was dichotomized into “younger” (18-20 years) and “older” (≥21) and weight status into “normal weight” (BMI < 25 kg m^−2^) and “overweight/obese” (BMI ≥ 25 kg m^−2^). Differences in readiness to control weight by weight status were investigated by including the group × weight status interaction in the ordinal regression model.

To assess the effect of risk status on motivation to control weight by genotype, *FTO* status was dichotomized into higher/lower risk, with those having at least one risk allele being classified as higher risk in accordance with previous studies 29,30. Ordinal regression analyses were used to examine effects of risk status on readiness to control weight by first comparing higher *FTO* risk and lower risk with controls and then comparing higher with lower risk. Age, gender, and baseline weight status were included as predictor variables in all models. To assess group differences in weight control behaviors, we built a mean score of the frequency of adherence to the tips included in the advice. Data were analyzed using ANCOVA including age, gender and weight status. Bonferroni corrections for multiple comparisons were used in all analyses, at *α* = 0.05.

## Results

### Participant flow and participant characteristics

Of 1,016 participants taking part at baseline (consenting, completing BMI measurement, and giving basic demographic information and a saliva sample for DNA analysis), 77 (7.5%) (intervention: *n* = 26; control *n* = 51) were excluded because their genotype could not be determined (Figure 1). Of the 939 participants invited to complete the motivation questionnaire 1 month after getting the *FTO* feedback (or matched time in controls), 383 (40.7%) completed it, just attaining the expected 40% completion rate. Participants who completed the questionnaire assessing readiness to control weight (vs. did not) were likely to be older, *t*(937) = −1.99, *P* = 0.046) and female (*χ*^2^(1) = 13.25, *P* < 0.001), and had lower BMI at baseline (*t*(937) = 2.77, *P* = 0.006). Drop-out was not related to group allocation (*P* = 0.317). Participants who reported having controlled their weight for more than 1 month were excluded from further analyses (27.2%, *n* = 104; FA = 59, AO = 45) because they already had reached the maintenance stage of weight control. These participants were more likely to be female (*χ*^2^ (1) = 9.14, *P* = 0.002), with slightly higher BMI at baseline (*t* (381) = −3.29, *P* = 0.001), and no differences in age. The final sample for analysis of the motivational effects of genetic test feedback therefore consisted of 279 participants. Participant characteristics for each randomized group are shown in Table2, and by genotype in Table3.

**Table 2:** Participant characteristics at follow-up

	Intervention (feedback and advice, *n* = 139)		Control (advice only, *n* = 140)	
**Gender, male % (n)**	51.1	(71)	47.9	(67)
**Age in years mean (SD)**	20.2	(2.5)	20.9	(3.0)
**Height in m, mean (SD)**	1.70	(0.1)	1.70	(0.1)
**Weight in kg, mean (SD)**	62.3	(10.8)	63.0	(11.7)
**BMI in kg m^−2^, mean (SD)**	21.2	(2.5)	21.4	(2.6)
**Normal weight, % (*n*) <25**	92.1	(128)	89.3	(125)
**Overweight/obese, % (*n*) ≥25**	7.9	(11)	10.7	(15)
***FTO* status, % (n)**				
**AA**	13.7	(19)	-	-
**AT**	39.6	(55)	-	-
**TT**	46.8	(65)	-	-

BMI: body mass index; TT: lower-risk *FTO* gene status; AT, AA: higher-risk *FTO* gene status.

**Table 3:** Participant characteristics at follow-up by genotype

	Control		TT		AT/AA	
**Gender, male % (n)**	47.9	(67)	47.7	(31)	54.1	(40)
**Age in years mean (SD)**	20.9	(3.1)	20.2	(2.7)	20.2	(2.7)
**Height in m, mean (SD)**	1.70	(0.09)	1.70	(0.1)	1.71	(0.09)
**Weight in kg, mean (SD)**	63.0	(11.7)	60.5	(11.8)	63.8	(11.8)
**BMI in kg m^−2^, mean (SD)**	21.5	(2.6)	20.7	(2.4)	21.7	(2.7)
**Normal weight, % (*n*) <25**	89.3	(125)	95.4	(62)	89.2	(66)
**Overweight/obese, % (*n*) ≥25**	10.7	(15)	4.6	(3)	10.8	(8)

BMI: body mass index; TT: lower-risk *FTO* gene status; AT, AA: higher-risk *FTO* gene status

### Primary outcome: Readiness to control weight at follow-up

Participants in the FA group were significantly more likely to be in the contemplation stage (thinking about controlling their weight) or the action stage (having started to control weight) than those in the control group (OR = 1.77, 95% CI = 1.08-2.89, *P* = 0.023) (see Table4), although the mean scores in both groups indicated low motivation overall (FA: 1.6, SD = 0.8; AO: 1.5, SD = 0.8).

**Table 4:** Ordinal logistic regression (PLUM) for the effect of the intervention (FA vs. AO) on readiness to control weight

	Multivariate model			Multivariate model including interaction terms		
Predictor variable	OR	95% CI	*P* value	OR	95% CI	*P* value
**Gender**						
**Male**	1			1		
**Female**	2.91	1.76-4.81	<0.001	2.98	1.79-4.95	<0.001
**Age**						
**18-20**	1			1		
**21-30**	0.87	0.53-1.44	0.594	0.89	0.53-1.48	0.646
**Weight status**						
**BMI < 25**	1			1		
**BMI ≥ 25**	4.80	2.14-10.77	<0.001	2.32	0.79-6.83	0.127
**Group**						
**AO**	1			1		
**FA**	1.77	1.08-2.89	0.023	1.46	0.87-2.45	0.127
**Intervention group × BMI < 25**				1		
**Intervention group × BMI ≥ 25**	-	-	-	6.67	1.13-39.25	0.036

BMI: body mass index; FA: feedback and advice group; AO: advice only

### Secondary outcomes

#### Effects of the intervention in subgroups at follow-up

Women were more likely to be in the contemplation stage than men (OR = 2.91, 95% CI = 1.76-4.81, *P* < 0.001). Overweight/obese participants were also more likely to be in the contemplation stage than those of normal weight (OR = 4.80, 95% CI = 2.13-10.77, *P* < 0.001).

**Figure 2:**
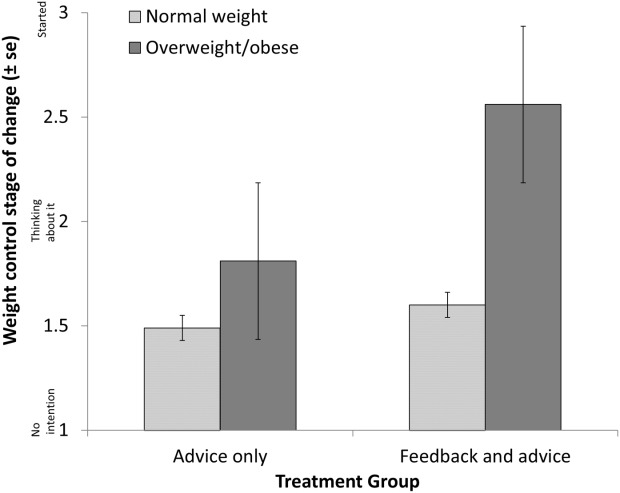
Effect of *FTO* genetic feedback on readiness to control weight in subgroups.

As shown in Figure 2, the group × weight status interaction was significant, with overweight/obese individuals in the FA group being more likely to be in the contemplation stage or the action stage at 1-month follow-up than normal-weight individuals in the FA group (OR = 6.67, 95% CI = 1.13-39.25, *P* = 0.036).

#### Effect of FTO risk status on readiness to control weight at follow-up

The *FTO* genotype was in Hardy–Weinberg equilibrium in the current sample (*χ*^2^ (2) = 5.68; *P* = 0.058). Nineteen (13.7%) participants had the higher-risk AA genotype, 55 (39.6%) had the intermediate risk AT genotype and 65 (46.8%) had the lower-risk TT genotype.

**Figure 3:**
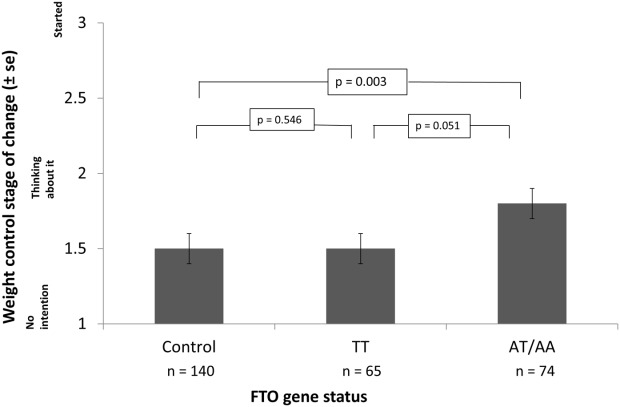
Readiness to control weight by *FTO* status.

As shown in Figure 3, there was a significant effect of *FTO* status on readiness to control weight at 1-month follow-up, with higher-risk participants being more likely to be in the contemplation stage than control participants who were in the precontemplation stage and had no weight control intentions (OR = 2.38, 95%CI = 1.33-4.26, *P* = 0.003). There was also a trend for higher risk (AT/AA) participants to be more likely to be in the contemplation stage than lower-risk (TT) participants who were in the precontemplation stage (OR = 1.97, 95%CI = 1.00-3.88, *P* = 0.052). There was no significant difference in readiness to control weight between lower-risk (TT) participants and those in the control group (*P* = 0.546), as shown in Table5.

**Table 5:** Ordinal regression analysis (PLUM) for the effect of *FTO* status (control vs. TT vs. AT/AA) on readiness to control weight

Predictor variable	OR	95% CI	*P* value
**Gender**			
**Male**	1		
**Female**	3.15	1.88-5.28	<0.001
**Age group**			
**18-20**	1		
**21-30**	0.96	0.39-2.36	0.360
**Weight status**			
**BMI < 25**	1		
**BMI ≥ 25**	4.90	2.17-11.07	<0.001
***FTO* feedback**			
**Control**	1		
**TT**	1.21	0.65-2.27	0.546
**AT, AA**	2.38	1.33-4.26	0.003
***FTO* feedback**			
**TT**	1		
**Control**	0.82	0.44-1.54	0.546
**AT, AA**	1.97	1.00-3.88	0.051

BMI: body mass index; TT: lower-risk *FTO* gene status; AT, AA: higher-risk *FTO* gene status.

#### Behavior change

A factor analysis confirmed that the individual items in the composite scale shared a common underlying structure (Crohnbach's *α* = 0.72). The overall number of tips followed was low (1.42, SD = 1.7), reflecting “occasional” use of weight control behaviors, and there was no significant difference in frequency between groups (*P* = 0.874). Age, gender and weight status also showed no association with the number of tips followed.

### Potential harms

We were not made aware of any harm caused by the intervention. In fact, participants made many positive comments, suggesting that both weight control advice and the genetic test feedback were well received.

## Discussion

This is the first study to investigate the utility of *FTO* genetic test feedback to motivate young, healthy individuals with weight control. In line with our hypothesis, weight control advice in conjunction with *FTO* feedback successfully increased motivation more than weight control advice alone, and effects were stronger in those receiving a higher-risk result. Importantly, lower-risk *FTO* feedback did not decrease motivation to engage with weight control, with effects being equivalent to receiving no genetic test feedback. This finding matches those from the smoking cessation field 31,32 and hints that complacency to lower-risk genetic test feedback for weight gain prevention may not be as much of a concern as has been thought. One reason may be that individuals may hold multifaceted causal explanations of weight gain, including both genetics and environmental factors 33, which may diminish any adverse impact of the genetic test result.

Genetic test feedback appeared to be especially effective in increasing readiness to prevent weight gain for individuals who were already overweight, perhaps because of greater perceived relevance of the test result. Alternatively, it is possible that genetic test feedback reduces self-blame and stigma, which has been posited as a barrier to weight control 7,26. Given that individuals did not enroll specifically in a weight control intervention, this is encouraging. However, the results have to be viewed with caution because only a small number of overweight/obese individuals returned for follow-up. Equally, although the intervention achieved modest effects on readiness to control weight, contrary to our secondary hypothesis, this did not translate into action, regardless of the gene test status. However, the results might plant a seed that could have effects in the future (e.g., if they gain weight). Long-term studies on the effects of genetic test feedback for common conditions are needed in this new field of research.

These findings add to the emerging literature on the effects of genetic test feedback as an aid for prevention and control of common, complex disorders. In contrast to earlier work focused on improving treatment adherence 34, the focus on prevention of weight gain in a community sample of young, healthy adults at university is novel. However, despite differences in context, our findings match earlier studies 10,22,32,34 in finding that genetic test feedback can affect behavior change intentions, shows no obvious adverse psychological effects, but has little or no effect on actual behavior change.

This study had a number of strengths. Despite high drop-out rates, it is one of the first trials to be powered to detect an effect of genetic test feedback on the outcome of interest. The study set-up provided a model for a possible “real world” scenario should genetic test feedback be introduced on a large scale to aid disease prevention, i.e., we chose a young, healthy population largely unaware of their genetic risk. The intervention could also be administered to a large sample without specific training, in a cost-effective manner (gene testing was priced at 1.50 pounds sterling per analysis), and without extensive staff resources.

It also had important limitations. Baseline weight control intentions were not assessed, so that no direct evidence of change in motivation as a result of genetic test feedback is available, only a between-group comparison (although groups were randomized). Although readiness to control weight was assessed with an established measure of stage of change, it comprised only a single item, which limits robustness of the findings. Future studies could explore the topic using more straightforward measures of motivation. Furthermore, since we were interested in the effect of *FTO* feedback on initiation of weight control, we had to exclude about a quarter of participants due to the chosen measure, although the sensitivity analysis indicated that effects were not significantly different when all participants were included. In addition, although the weight control leaflet was evidence-based, it had not been piloted specifically in the prevention context. The absence of a “no treatment” control group precluded any conclusion on whether the leaflet alone would be effective in behavior change, but that was not the focus of the study. Participants were not specifically encouraged to follow the tips in the leaflet because we were interested in whether genetic test feedback would be a sufficient prompt for initiating action without additional support. They may have engaged in alternative weight control behaviors not mentioned in the tips, but these were not assessed. Finally, the study suffered from high drop-out rates. Although this was anticipated, it nonetheless limits the generalizability of findings. Participants were more likely to enroll if they were normal weight and less likely to return for follow-up if they had a higher BMI at baseline, which limited opportunities for exploring effects of *FTO* test feedback in individuals who were already overweight.

## Conclusion

This study provides evidence that *FTO* genetic test feedback can successfully increase readiness to control weight in a young, healthy population in a situation with established risk of weight gain, but it found no evidence that it impacted actual behavior. However, importantly, it did not lessen weight control intentions or behaviors, suggesting that concerns about genetic determinism leading to disengagement from behavior change following obesity genetic testing may be unfounded.
